# Impact of Prolonged Cycle Length Resulting From Conversion of Atrial Fibrillation to Atrial Tachycardia on Ablation Outcome in Persistent Atrial Fibrillation Ablation

**DOI:** 10.1155/2024/8880826

**Published:** 2024-06-08

**Authors:** Jialing He, Guoshu Yang, Duan Luo, Yongxin Yang, Guijun He, Xianchen Yang, Zhen Zhang

**Affiliations:** ^1^ Department of Cardiology The Affiliated Hospital of Southwest Jiaotong University The Third People's Hospital of Chengdu Cardiovascular Disease Research Institute of Chengdu, Chengdu, Sichuan, China; ^2^ Physical Examination Department Modern Hospital of Sichuan, Chengdu, Sichuan, China

**Keywords:** atrial fibrillation, catheter ablation, cycle length

## Abstract

**Background:** There is limited available data regarding the impact of cycle length (CL) prolongation when converting atrial fibrillation (AF) to organized atrial tachycardia (AT) and its effect on clinical outcomes.

**Methods and Method:** We retrospectively screened and included a cohort of 132 patients with persistent or long-standing persistent AF who underwent circumferential pulmonary vein isolation (CPVI) and left atrial substrate modification (LASM) between January 2015 and October 2019. In all 132 consecutive patients, persistent AF was successfully converted into organized AT. For cases with recurrence after a 3-month blanking period, a repeat procedure was conducted.

**Results:** We observed a notable prolongation in CL after ablation (average increase of 56.6 ± 30 ms). Following a median follow-up duration of 9.5 ± 5.1 months, 27 patients experienced recurrence. Through receiver operating curve (ROC) analysis, a prolonged CL cut-off of 42.5 ms was identified, with a specificity of 71% and a sensitivity of 59.4%. Patients were categorized into two groups: those with CL less than 42.5 ms (group A, *n* = 48) and those with CL more than 42.5 ms (group B, *n* = 84). The Kaplan–Meier survival curves demonstrated a significantly higher recurrence-free rate after catheter ablation in group B compared to group A (*p* = 0.002).

**Conclusions:** Upon termination of persistent AF into AT during ablation, it was found that CL prolongation beyond 42.5 ms was associated with improved freedom from arrhythmia.

## 1. Background

Atrial fibrillation (AF) is the most common sustained cardiac arrhythmia [1]. Persistent AF is associated with a higher AF burden and a higher stroke and mortality risk than paroxysmal AF [2]. However, the aggregate evidence for the efficacy of catheter ablation for persistent AF is weaker than that of paroxysmal AF [3]. It was reported that 18 months after persistent AF radiofrequency ablation, AF recurrence of different kinds of ablation strategies was about 50%, even in the most experienced centers, requiring a mean of 1.8 procedures/patient [4, 5]. So radiofrequency catheter ablation of persistent or long-standing persistent AF is still challenging. Pulmonary vein (PV) isolation is the cornerstone of AF ablation. Building upon PV isolation, low-voltage area ablation has been proven to be an effective strategy for persistent AF ablation [6, 7].

During the ablation procedure, with extensive ablation, persistent AF was usually converted directly into sinus rhythm or organized atrial tachycardia (AT) which required further ablation or direct current (DC) cardioversion. AF converted to organized AT was a dominant termination mode during the ablation [5, 8–10]. Sometimes AT was difficult to delineate by an electroanatomic mapping system. Moreover, further ablation to terminate the AT required longer procedure time, fluoroscopy time, and a higher risk of collateral damage. Significant cycle length (CL) prolongation during ablation was frequently reported, and it may be related to better ablation outcomes [11–13]. We conducted this study intending to figure out the value of CL prolongation on outcome while AF was converted to AT during ablation using a circumferential PVI plus low voltage area ablation strategy.

## 2. Material and Methods

### 2.1. Study Population

Consecutive patients in our center from January 2015 to October 2019 with persistent/long-standing persistent AF who underwent catheter ablation and consented to participate were screened for this retrospective study. Persistent AF was defined as an AF episode either lasting longer than 7 days or requiring termination by cardioversion, and long-standing persistent AF should last for more than 1 year. Antiarrhythmic drugs, except amiodarone, were ceased at least 5 half-lives before the ablation. The patients who had undergone a prior ablation of AF were excluded. All patients gave signed informed consent.

### 2.2. Electrophysiological Study

All patients received warfarin therapy (target international normalized ratio (INR): 2–3) or NOAC for at least 1 month before the procedure. Anticoagulation was continued with low-molecular-weight heparin following warfarin discontinuation. Transesophageal echocardiography was performed within 48 h to exclude the left atrial thrombi.

Surface electrocardiograms and endocardial electrograms were continuously monitored and stored for further study (bipolar recordings were filtered from 30 to 500 Hz). One decapolar catheter (Biosense Webster) was positioned into the coronary sinus via the left subclavian vein for recording left atrial electrical activity and for pacing. One mapping catheter (PentaRay, Biosense Webster) was positioned in the PV ostium to map pulmonary vein potentials (PVPs) and used for left atrial substrate (LAS) mapping. The 3.5-mm saline-irrigated tip ablation catheter (Thermocool NaviStar, Biosense Webster) was used for ablation. A deflectable sheath (after transseptal puncture) was used to stabilize the PentaRay and ablation catheter. Heparin (100 U/kg of body weight) was injected and followed by 1000 U/h to maintain the activated clotting time (ACT) between 300 and 350 s.

### 2.3. Circumferential Pulmonary Vein Isolation (CPVI) and Left Atrial Substrate Modification (LASM)

Selective retrograde PV venography was performed to identify the ostia of PVs. Under the guidance of the electroanatomical mapping system (Carto, Biosense Webster), the left atrium anatomy was reconstructed and each PV ostium was identified and tagged on the electroanatomical map. Contiguous application of irrigated radiofrequency energy was delivered with 50 W guided by AI, and all points were ensured adequate catheter tissue contact by the contact force (5–10 g). The AI cutoff values varied with location: anterior wall, 450–500; posterior wall, 380; and roof, 400. Ablation duration was guided by AI and mainly fluctuated between 8 and 20 s. The saline infusion rate was set at 17 mL/min. Ablation was performed at the PV antrum (5 mm away from the angiographically defined PV ostia). Successful CPVI was demonstrated by PVP disappearance or left atrium-PV potential dissociation. LAS and electroanatomic voltage mapping were performed before the CPVI. Substantially (< 0.5 mV) reduced voltage areas of the left atrium were detected by electroanatomic voltage mapping through the Carto system. Areas of substantial fibrosis are defined as substantially reduced voltage areas (< 0.5 mV) of the left atrium, and LASM is defined as homogenization ablation of the substantial fibrotic region. After the isolation of each PV, homogenization ablation was applied to achieve homogenization of the areas of substantial fibrosis. The ablation catheter was navigated using a point-by-point approach. In instances meeting this criterion, both entrance and exit blocks were verified between the posterior wall and other regions of the left atrium. Furthermore, caution was exercised to prevent LVA ablation in zones that might lead to or trigger conduction disturbances. The endpoint of LASM ablation was the electrostatic zone (< 0.1 mV) of the substantial fibrotic region.

### 2.4. CL Measurement and Procedural Endpoint

Before the ablation and after the AF converted into AT, the CL was measured for all patients. CL was determined within the coronary sinus by averaging 10 consecutive cycles using automated monitoring software. AT was defined as an organized atrial rhythm with a stable CL and activation sequence. No additional ablation was used to terminate the AT, and DC cardioversion was performed to restore sinus rhythm.

### 2.5. Postablation Management and Follow-Up

During hospitalization, the ECG was continuously monitored, low-molecular-weight heparin was injected ≧ 4 days, and warfarin therapy was instituted to keep the INR in the therapeutic range of 2 to 3. A Class III antiarrhythmic drug of amiodarone, 600 mg/day, was administered in all patients and was discontinued after 3 months (blanking period) in cases of recurrence. After hospital discharge, all patients were followed for clinical evaluation. Surface ECG and 24-h Holter recording were performed at 1, 3, 6, 12, 18, and 24 months postprocedure. A monthly telephone inquiry was applied to evaluate the symptom recurrence during the follow-up visits. When the patients experienced symptoms indicating arrhythmias after the catheter ablation, a surface ECG and 24-h Holter recording were performed to define the cause of the clinical symptoms. The primary endpoint was atrial arrhythmia recurrence, including AF, atrial flutter, and AT. Recurrence was defined as an episode of symptomatic or asymptomatic atrial arrhythmias lasting > 30 s documented by Holter monitoring and surface ECG. All recurrences after the blanking period (3 months) were considered failures. Repeat ablation was advised for all cases that experienced recurrence after the blanking period. Success was defined as the absence of all documented symptomatic or asymptomatic atrial arrhythmias without antiarrhythmic drugs.

### 2.6. Statistical Analysis

All continuous variables were expressed as mean ± standard deviation and compared by Student's test. Categorical variables were reported as counts or proportions and compared by chi-square or Fisher's exact test. Receiver operating curves (ROC) were analyzed, and the area under the curve was calculated to determine a specific CL prolongation cut-off that predicted the outcome postablation. The optimal cutoff point was chosen as the combination with the highest sensitivity and specificity. Freedom of recurrence analysis of CL prolongation was performed using log-rank tests with the assistance of Kaplan–Meier curves. All tests were two-tailed, and statistical significance was established at *p* < 0.05. Statistical analysis was performed with SPSS for Windows (version 12.0.1).

## 3. Results

### 3.1. Patient Population

A total of 734 patients underwent AF ablation. Out of these, 510 patients were unable to terminate AF, while 52 patients achieved direct sinus rhythm. Additionally, 40 patients initially converted to AT and subsequently underwent ablation to achieve sinus rhythm. Furthermore, 132 patients experienced a conversion to AT, which could not be terminated, and were finally cardioverted to restore SR by DC cardioversion. The reason for the inability to surgically terminate AT was due to the inability to accurately map this group of patients. These 132 patients were our study population. The overall grouping of patients is shown in Figure S1.

None of the patients had received ablation before. The characteristics of all patients are shown in Table 1. The whole 132 patients had an average age of 63.2 ± 9.6 years, with 84 males (63.6%). The median AF duration was 3.2 ± 4.1 years. The baseline and prolonged CL were 166.6 ± 17.6 and 56.6 ± 30 ms, respectively. There were no significant differences in any of the assessed baseline characteristics. Patients were followed up for a median of 9.0 ± 6.8 months after the final ablation procedure. At the end of the follow-up, 27 patients had a recurrence.

All patients underwent AF conversion into organized AT, which was subsequently restored to sinus rhythm through cardioversion. The sites of AF conversion into AT were located at the coronary sinus in 12 patients, left atrial roof in 21, septum in 25, mitral isthmus in 14, left atrial appendage in 27, left anterior wall in 27, and lateral left atrium in 6. No antiarrhythmic drugs were used before DC cardioversion. Sinus rhythm was restored by DC cardioversion in all of the patients.

### 3.2. CL Prolongation and Subgroup Analysis

**Figure 1:**
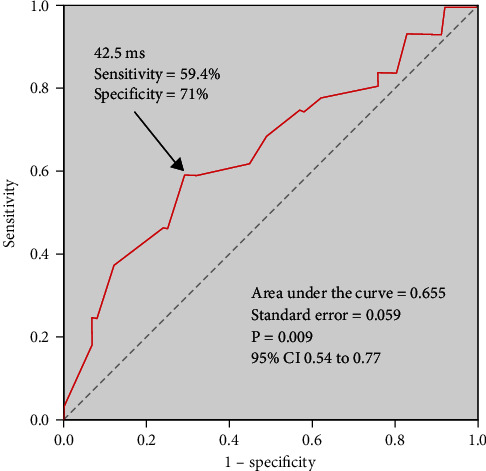
The receiver-operator characteristic (ROC) curve analysis. Arrow indicates optimal cutoff point for sensitivity and specificity.

Progressive prolongation of the baseline AFCL was observed during the process (baseline CL 166.6 ± 17.6 ms vs. postablation CL 224.0 ± 37.8 ms, *p* < 0.001). The relationship between the prolongation of CL when AF was converted into AT and procedural success was analyzed with ROC. The ROC indicated an optimal cutoff point of 42.5 ms with an area under the curve of 0.655 (95% CI, 0.54 to 0.77, *p* = 0.009) (shown in Figure 1). The specificity and sensitivity were 71% and 59.4%, respectively, in predicting the clinical outcome. CL prolongation of > 42.5 ms was found to have the highest accuracy for predicting long-term success.

Then, we separated patients into two groups depending on CL prolongation degree. Patients with CL prolongation of less than 42.5 ms were divided into group A, and those with CL prolongation of more than 42.5 ms were in group B. In total, 48 patients were divided into group A, and the other 84 patients were in group B. The characteristics of these patients are listed in Table 1. There were no differences in age, gender, complications, LA diameter, left ventricular ejection fraction, left ventricular diastolic end diameter, and AF duration between the two groups. The baseline AFCL of group A and group B was 164.1 ± 16.9 ms versus 168.1 ± 18.40 ms (*p* = 0.26). When AF converted into AT, prior to DC cardioversion, the CL prolonged to 191.5 ± 19.0 ms versus 243.4 ± 32.5 ms (*p* < 0.0001), with prolongation of 27.7 ± 6.9 and 73.1 ± 25.2 ms, respectively (*p* < 0.001). There were 31 (69.5%) patients free from arrhythmia in group A and 74 (88.1%) patients free from arrhythmia in group B (*p* = 0.003).

**Figure 2:**
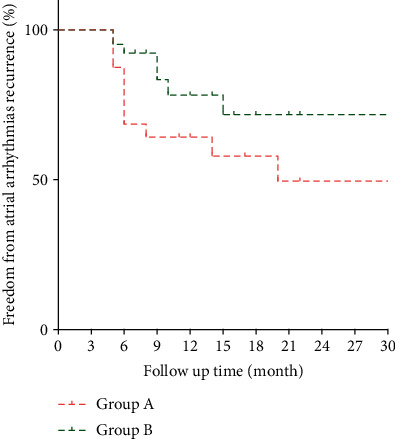
Kaplan–Meier curve of freedom from recurrence.

Based on the observation of better outcomes in group B over group A, we depicted the Kaplan–Meier survival curve to express freedom from arrhythmia, which is shown in Figure 2. After the final procedure, freedom of recurrence after catheter ablation was significantly higher in group B compared with group A when AF was converted into organized AT (*p* = 0.002).

## 4. Discussion

Progressive prolongation of CL was observed when AF converted into organized AT, and according to ROC analysis, the cut-off prolonged CL should be 42.5 ms. The Kaplan–Meier survival curves indicated that patients with CL prolongation of more than 42.5 ms had better outcomes with less atrial arrhythmia recurrence.

While CL prolongation was frequently observed during ablation for persistent AF, only a limited number of studies have investigated its predictive significance. For instance, Kochhäuser et al. conducted a substudy of the STAR AF II trial. They established a CL prolongation cut-off of 20 ms and concluded that this prolongation did not significantly impact freedom from AF when compared to patients without CL prolongation (48% vs. 45%; *p* = 0.83) [14].

By targeting critical areas that sustain AF and altering the atrial substrate, AF can occasionally transit directly into sinus rhythm or organized AT. Past research has highlighted that the predominant mode of termination for AF is conversion into organized AT rather than direct conversion into sinus rhythm [5, 9, 10]. Left and right atrium and even the coronary sinus were often necessary to terminate the AT and restore sinus rhythm. It is worth noting that progressive prolongation of AFCL is typically observed during ablation procedures in patients with persistent AF.

Patients with persistent AF often exhibit characteristics such as a larger left atrium diameter, a lower mean atrial voltage, increased refractoriness heterogeneity, and shorter AFCLs [15, 16]. This arrhythmia-related remodeling process is believed to underlie the substrate of persistent AF. Haissaguerre et al. proved that atrial fibrillatory CL was inversely associated with the number of sources participating in AF maintenance [17]. Consequently, in clinical practice, shorter AFCLs require more extensive ablation to target sources for atrial substrate modification and AF termination compared to longer AFCLs. Once a critical AFCL, typically over 200 ms, is achieved, AF can no longer be maintained, leading to conversion into SR or AT. Although, in most cases, AT becomes the dominant mode of AF termination, its complexity poses challenges for mapping the mechanism. Further ablation to terminate AT can result in prolonged procedures and fluoroscopy times, along with potential complications like pericardial tamponade or nerve injuries. Inadequate linear lesions to terminate AT might result in gap-related reentrant AT. At last, DC cardioversion had to be performed to convert the AT into SR in some patients with failed ablation to terminate the AT.

When AF is converted into organized AT, varying degrees of CL prolongation are observed. However, the necessity of ablating all converted organized ATs with different CL prolongation levels and their connection to clinical outcomes has not been assessed in prior studies. In this study, the hypothesis is that a higher degree of CL prolongation during the conversion from AF to AT might indicate successful modification of the abnormal atrial substrate, potentially leading to favorable clinical outcomes. Patients with differing degrees of CL prolongation during the AF to AT conversion were divided into two groups (groups A and B) using sensitivity and specificity statistical analysis. When CL prolongation reaches an optimal point, typically exceeding 42.5 ms, DC cardioversion could be a viable option to restore sinus rhythm, minimizing risks like collateral damage, prolonged fluoroscopy, and procedure times.

### 4.1. Study Limitations

This was a retrospective single-center study, and the data might only apply to the procedure in which CPVI combined with the LASM ablation strategy was performed. In this study, we only analyzed the CL prolongation and its impact on the clinical outcome. The mechanism of AT, reentrant, or focal tachycardia, with different degrees of CL prolongation and its impact, needed further study to assess. There were asymptomatic AF recurrences which might have been missed, although all patients underwent regular clinical interviews for follow-up through a 24-h Holter and frequent ECG examinations. Certainly, given the limited sample size of this study, further prospective studies are essential to validate and reinforce these findings.

## 5. Conclusion

Our study observed a noteworthy increase in CL when AF was converted to AT during ablation. CL prolongation exceeding 42.5 ms was associated with improved freedom from arrhythmia.

## Figures and Tables

**Table 1:** Comparison of baseline characteristics in patients.

**Characteristics**	**All population (** **n** = 132**)**	**Group A (** **n** = 48**)**	**Group B (** **n** = 84**)**	**p** **value**
Male	84 (63.6%)	29	55	0.59
Age	63.2 ± 9.6	62.7 ± 9.3	63.5 ± 9.7	0.65
Hypertension	43 (32.6%)	13 (27.1%)	30 (35.7%)	0.34
Diabetes mellitus	11 (8.3%)	3 (6.2%)	8 (9.5%)	0.74
Congestive heart disease	6 (4.5%)	1 (2.1%)	5 (5.9%)	0.31
Rheumatic heart disease	9 (6.8%)	1 (2.1%)	8 (9.5%)	0.20
Stroke	9 (6.8%)	2 (4.2%)	7 (83.3%)	0.36
AF during (years)	3.2 ± 4.1	2.7 ± 2.5	3.5 ± 4.7	0.22
LA diameter (mm)	44.9 ± 4.6	44.4 ± 4.7	45.2 ± 4.6	0.33
LA volume	44.9 ± 4.6	142.3 ± 32.0	143.6 ± 35.7	0.80
LV diastolic diameter	49 ± 4.9	50.3 ± 7.4	48.6 ± 4.5	0.06
Ejection fraction (%)	57 ± 6.4	56.3 ± 7.4	58.5 ± 5.7	0.06
Baseline CL (ms)	166.6 ± 17.6	164.1 ± 16.9	168.1 ± 18.0	0.26
Post-CL (ms)	224.0 ± 37.8	191.5 ± 19	243.4 ± 32.5	< 0.001
CL prolongation (ms)	56.6 ± 30	27.7 ± 6.9	73.1 ± 25.2	< 0.001
Follow-up duration	9.0 ± 6.9	9.7 ± 7.8	8.8 ± 6.3	0.39
Repeat ablation	20 (15.2%)	8 (16.7%)	12 (14.3%)	0.71
Recurrence	27 (20.5%)	17 (35.4%)	10 (11.9%)	0.003
Repeat ablation recurrence	1 (0.08%)	1 (2%)	0 (0.0%)	0.18
Recurrence type				
AF	11 (0.8%)	7 (14.6%)	3 (3.6%)	< 0.001
AT	16 (12.1%)	10 (20.8%)	6 (7.1%)	0.02

*Note:* Group A: patients with CL prolongation less than 42.5 ms. Group B: patients with CL prolongation more than 42.5 ms.

## Data Availability

Data is available on request from the authors.
