# Supplementary figures and images for: Impact of Prolonged Cycle Length Resulting From Conversion of Atrial Fibrillation to Atrial Tachycardia on Ablation Outcome in Persistent Atrial Fibrillation Ablation
Source: Cardiovasc Ther. 2024 Jun 8;2024:8880826. doi: 10.1155/2024/8880826 (PMC11221985; doi:10.1155/2024/8880826)

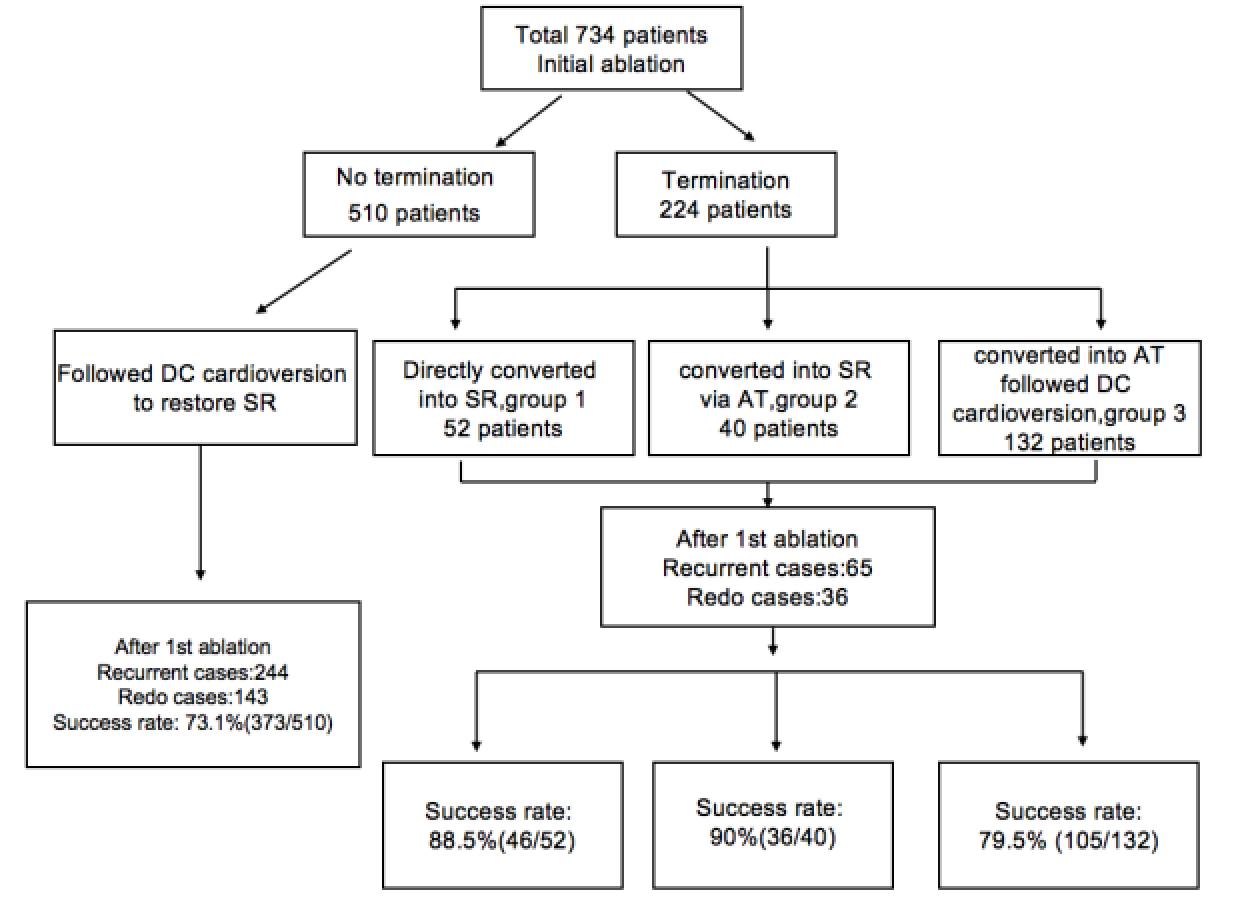

Supplement: Supporting Information — Additional supporting information can be found online in the Supporting Information section. Figure S1 Patients flow chart. [file 8880826.f1.jpg]
